# Recurrence after postoperative intravesical instillation therapy in Hunner type interstitial cystitis

**DOI:** 10.1038/s41598-023-44894-x

**Published:** 2023-10-25

**Authors:** Kwang Jin Ko, Michael Jakun Koo, Seokhwan Bang, Hye Jin Byun, Min-Ji Kim, Kyunga Kim, Kyu-Sung Lee

**Affiliations:** 1 Department of Urology, Samsung Medical Center, Sungkyunkwan University School of Medicine, 81 Irwon-ro, Gangnam-gu, Seoul, 06351 Korea; 2 Department of Urology, Seoul St. Mary’s Hospital, College of Medicine, The Catholic University of Korea, Seoul, Korea; 3 Department of Urology, Dongsan Medical Center, Keimyung University School of Medicine, Daegu, Korea; 4 Biomedical Statistics Center, Research Institute for Future Medicine, Samsung Medical Center, Seoul, Korea; 5 Department of Digital Health, SAIHST (Samsung Advanced Institute for Health Sciences & Technology), Sungkyunkwan University, Seoul, Korea; 6 Department of Data Convergence & Future Medicine, Sungkyunkwan School of Medicine, Seoul, Korea; 7 Research Institute for Future Medicine Samsung Medical Center, Seoul, Korea

**Keywords:** Diseases, Urology

## Abstract

We performed a prospective, single-arm study comparing outcomes between transurethral ablation plus postoperative instillation of hyaluronic acid and chondroitin sulfate (HACS group) and transurethral ablation only in patients with Hunner type interstitial cystitis (historical control group). A total of 78 patients were enrolled, and 51 were included in the per-protocol analysis set. The 2-year recurrence rate was 47.1% (95% CI, 32.9–61.5) in the HACS group, which was significantly lower than that in the control group (86.2%; 95% CI, 74.6–93.9, *P* < 0.001). After instillation therapy, the hazard ratio for recurrence was 0.38 (95% CI, 0.23–0.65, *P* < 0.001). The HACS group had an increased recurrence-free survival with the median interval not being reached, while it was 11.4 months in the control group (95% CI, 8.8–13.8, *P* < 0.001). Regardless of the instillation treatment, there were significant improvements in all symptom questionnaire scores and pain compared to the baseline. However, in the instillation group, improvement was stable even after 12 months. In patients with Hunner type interstitial cystitis, intravesical instillation of hyaluronic acid and chondroitin sulfate after transurethral ablation significantly reduced the recurrence rate and maintained symptom improvement for more than 1 year.

## Introduction

Interstitial cystitis/bladder pain syndrome (IC/BPS) is a chronic inflammatory condition of the bladder^1,2^. IC/BPS is associated with several disabling symptoms, such as decreased work productivity and mobility, sleep disorders, and sexual dysfunction due to chronic pain, that negatively impact the lifestyle of patients^3,4^. IC/BPS is categorized into the IC/BPS with Hunner lesions (Hunner type IC) and IC/BPS without Hunner lesions^1,2,5^. Hunner lesion is an abnormal mucosal finding in the bladder confirmed through cystoscopy and described as a circumscript, reddened mucosal area with small vessels radiating towards a central scar, with a fibrin deposit or coagulum attached to this area^6^. The histopathologic characteristics of Hunner lesions include chronic inflammation of the bladder mucosa, epithelial denudation and subepithelial mastocytosis^7–9^. Hunner type IC represents a distinct disease process that requires different management strategies^10,11^. Intravesical instillation treatments, such as dimethyl sulfoxide (DMSO) and glycosaminoglycan (GAG) substitution, are used for repairing the damaged bladder tissue by applying the agent directly^12^. In a systematic review, GAG substitution instillation was found to be a valuable option with limited evidence^13^. Another option is endoscopic ablation of the Hunner lesion directly. After ablation of the Hunner lesion, pain-related symptoms improved significantly, but the efficacy was not long-lasting due to the 2-year recurrence rate was as high as 75%^14,15^. Finally, when all other therapies have failed, approximately 10% of patients are considered for augmentation cystoplasty with partial cystectomy or complete cystectomy with urinary diversion^2,5,16^.

We hypothesized that intravesical instillation of hyaluronic acid and chondroitin sulfate (HACS), a GAG substitute, following transurethral ablation of Hunner lesions may reduce recurrence and prolong recurrence-free time compared to transurethral ablation alone.

## Results

### Patient demographics

Patients were enrolled between September 2017 and October 2019, and a 2-year follow-up was concluded in November 2021. A total of 78 patients were enrolled, of whom three did not undergo surgery or withdrew consent prior to surgery, 10 withdrew consent after surgery, two discontinued the instillation of HACS, and one was diagnosed with bladder cancer. Therefore, a total of 62 patients were included in the full analysis (FA) set. After completing the HACS treatment, 11 patients failed to undergo 2 years of follow-up and 51 patients were subjected to the per-protocol analysis (PPA) set. The baseline characteristics were well-balanced between the HACS and control groups (Table 1). In the FA set, the PUF symptom scale score was lower in the HACS group than in the control group (14.05 vs 15.53, *P* = 0.044); otherwise, the groups were comparable.

**Table 1:** Baseline characteristics*.

	Full analysis set			Per-protocol set		
	Control group (N = 69)	HACS group (N = 62)	*P*	Control group (n = 58)	HACS group (n = 51)	*P*
Age (yr)	62.15 ± 9.61	64.15 ± 9.92	0.024	61.98 ± 9.62	65.52 ± 8.92	0.050
Sex, n (%)						
Male	15 (21.7)	16 (25.8)	0.585	14 (24.1)	12 (23.5)	0.941
Female	54 (78.3)	46 (74.2)		44 (75.9)	39 (76.5)	
BMI (kg/m^2^)	23.19 ± 2.81	23.44 ± 2.65	0.569	23.04 ± 2.79	23.46 ± 2.58	0.315
O’Leary–Sant Interstitial Cystitis questionnaire						
Symptom index	16.07 ± 3.25	15.37 ± 3.98	0.482	15.93 ± 3.22	15.55 ± 3.98	0.988
Problem index	13.66 ± 2.47	13.42 ± 3.14	0.994	13.44 ± 2.56	13.51 ± 3.00	0.620
Pelvic pain and urgency/frequency						
Symptom score	15.53 ± 3.11	14.05 ± 3.86	0.044	15.21 ± 2.98	14.14 ± 3.88	0.109
Bothersome score	7.83 ± 1.84	7.18 ± 2.35	0.287	7.77 ± 1.9	7.27 ± 2.27	0.564
Pain for VAS	6.97 ± 1.86	7.03 ± 2.10	0.780	6.96 ± 1.78	7.31 ± 2.01	0.259
Voiding diary						
Daytime frequency	14.11 ± 7.64	13.88 ± 6.31	0.740	13.56 ± 7.36	13.86 ± 6.54	0.536
Nocturia	3.77 ± 2.50	3.16 ± 2.02	0.128	3.74 ± 2.62	3.11 ± 1.88	0.222
Urgency	12.99 ± 12.05	10.3 ± 8.75	0.377	12.5 ± 11.66	10.41 ± 8.89	0.570

*BMI* body mass index; *HACS* hyaluronic acid and chondroitin sulfate; *VAS* visual analog scale.

*Values are expressed as mean ± standard deviation.

### Recurrence rate and recurrence-free time

The 2- year recurrence rate was 47.1% (95% CI, 32.9–61.5) in the HACS group and 86.2% (95% CI, 74.6–93.9) in the control group (*P* < 0.001). In the multivariable logistic regression model, only HACS instillation (odds ratio, 0.13; 95% CI, 0.05–0.35, *P* < 0.001) was associated with a lower proportion of recurrence (Table 2).

**Table 2:** Multivariable logistic regression analysis for recurrence.

Covariables	Odds ratio (95% CI)	*P* value
HACS treatment	0.13 (0.05–0.35)	< 0.001
Age	1.01 (0.96–1.06)	0.843
Sex (Male vs Female)	1.27 (0.41–3.89)	0.679
PUF symptom scale	1.00 (0.88–1.15)	0.953
Number of Hunner lesions (> 1 lesion)	0.75 (0.24–2.33)	0.620

*CI* confidence interval; *HACS* hyaluronic acid and chondroitin sulfate; *PUF* Pelvic pain and urgency/frequency.

**Figure 1:**
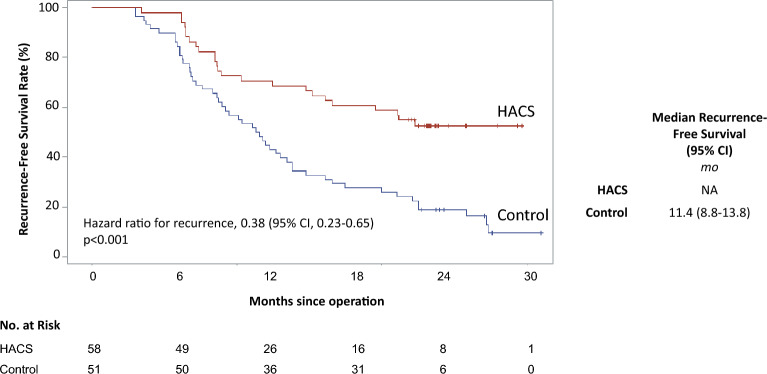
Recurrence-free survival curve estimated by the Kaplan–Meier method.

The median follow-up time for the HACS group was 23.3 months (95% CI, 23.1–23.7) and for control group was 27.6 months (95% CI, 23.7–31.0). The HACS group had increased recurrence-free survival with the median interval not reached. In contrast, the control group had a median survival of 11.4 months (95% CI, 8.8–13.8, *P* < 0.001) (Fig. 1). After adjusting the effect of confounders, the risk of recurrence was 61.7% lower in the HACS group than in control group (hazard ratio, 0.38; 95% CI, 0.23–0.65; *P* < 0.001) (Table 3).

**Table 3:** Cox regression for factors associated with recurrence of hunner type interstitial cystitis.

		Univariable analysis		Multivariable analysis*	
Set	Factor	HR (95% CI)	*P* value	HR (95% CI)	*P* value
PPA set	HACS instillation	0.39 (0.24–0.64)	< 0.001	0.38 (0.23–0.65)	< 0.001
FA set	HACS instillation	0.41 (0.26–0.67)	< 0.001	0.41 (0.25–0.69)	< 0.001

*FA* full analysis; *HACS* hyaluronic acid and chondroitin sulfate; *HR* hazard ratio; *PPA* per-protocol analysis.

*After adjusting for age, sex, number of Hunner lesions, pelvic pain, urgency/frequency symptom score, interaction of time, and symptom score.

O’Leary-Sant interstitial cystitis questionnaire, pelvic pain and urgency/frequency (PUF) and Pain Severity.

**Figure 2:**
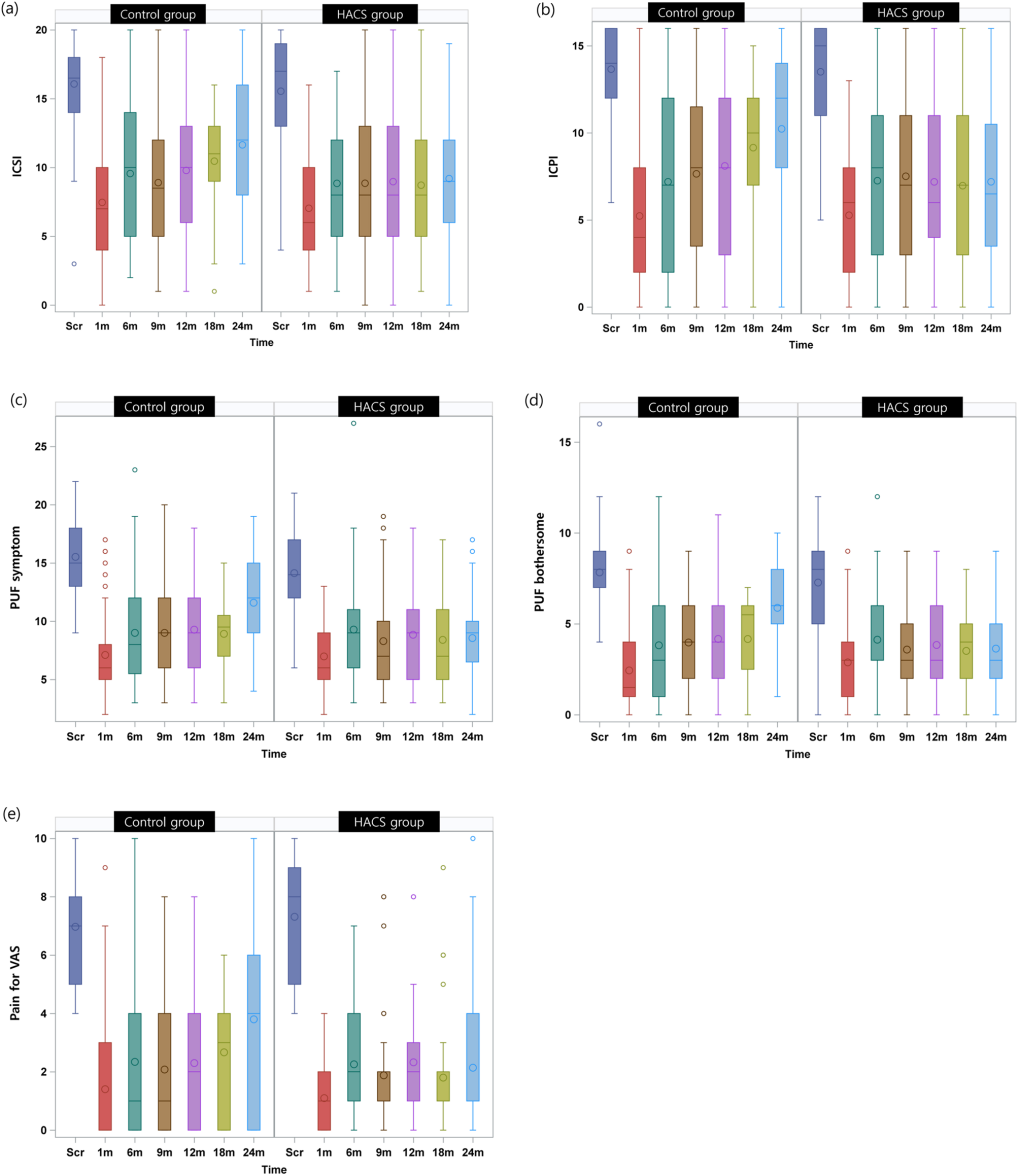
Changes in questionnaire and visual analogue scale for pain after treatment at each follow-up.

Both groups showed the greatest improvement at 1 month postoperatively in terms of visual analogue scale (VAS) for pain, O’Leary-Sant interstitial cystitis symptom index (ICSI) and problem index (ICPI), PUF, and showed a tendency to gradually deteriorate, but there was significant improvement compared to baseline at 24 months. Interestingly, VAS for pain, ICSI, ICPI, and PUF continued to deteriorate in the control group, whereas the HACS group maintained a similar level from 6 to 24 months (Table 4 and Fig. 2).

**Table 4:** Changes in O’Leary–Sant interstitial cystitis questionnaire scores, pelvic pain and urgency/frequency symptom scale, and visual analog scale score for pain after treatment between the groups at each study visit (Per-Protocol Set).

	Control group	HACS group	*P*^†^
ICSI			
At 1mo			
n	57	51	
Mean change from baseline	− 8.79 ± 4.55	− 8.51 ± 4.66	0.753
P*	< 0.001	< 0.001	
At 6mo			
n	48	39	
Mean change from baseline	− 5.77 ± 5.02	− 6.74 ± 5.14	0.377
P*	< 0.001	< 0.001	
At 9mo			
n			
Mean change from baseline	− 6.68 ± 4.96	− 6.44 ± 5.20	0.841
P*	< 0.001	< 0.001	
12mo			
n	34	41	
Mean change from baseline	− 5.84 ± 4.38	− 6.19 ± 5.51	0.763
P*	< 0.001	< 0.001	
18mo			
n	38	37	
Mean change from baseline	− 5.5 ± 4.56	− 6.34 ± 5.68	0.486
P*	< 0.001	< 0.001	
24mo			
n	12	35	
Mean change from baseline	− 4.73 ± 5.23	− 5.89 ± 5.90	0.514
P*	0.004	< 0.001	
ICPI			
1mo			
n	57	51	
Mean change from baseline	− 8.28 ± 4.28	− 8.24 ± 4.55	0.993
P*	< 0.001	< 0.001	
6mo			
n	48	39	
Mean change from baseline	− 5.65 ± 5.16	− 6.44 ± 5.33	0.392
P*	< 0.001	< 0.001	
9mo			
n	34	41	
Mean change from baseline	− 5.38 ± 4.81	− 5.80 ± 4.93	0.710
P*	< 0.001	< 0.001	
12mo			
n	38	37	
Mean change from baseline	− 4.82 ± 4.86	− 5.81 ± 5.47	0.407
P*	< 0.001	< 0.001	
18mo			
n	12	35	
Mean change from baseline	− 4.42 ± 4.74	− 6.20 ± 5.53	0.276
P*	0.002	< 0.001	
24mo			
n	15	36	
Mean change from baseline	− 4.00 ± 5.58	− 6.08 ± 5.70	0.238
P*	0.015	< 0.001	
PUF Symptom score			
1mo			
n	56	51	
Mean change from baseline	− 8.18 ± 3.98	− 7.16 ± 4.26	0.203
P*	< 0.001	< 0.001	
6mo			
n	47	39	
Mean change from baseline	− 5.60 ± 4.61	− 5.08 ± 5.73	0.643
P*	< 0.001	< 0.001	
9mo			
n	33	41	
Mean change from baseline	− 5.64 ± 4.24	− 6.00 ± 5.01	0.741
P*	< 0.001	< 0.001	
12mo			
n	38	37	
Mean change from baseline	− 5.76 ± 4.12	− 4.70 ± 5.08	0.323
P*	< 0.001	< 0.001	
18mo			
n	11	35	
Mean change from baseline	− 6.00 ± 3.97	− 5.40 ± 5.15	0.725
P*	< 0.001	< 0.001	
24mo			
n	15	36	
Mean change from baseline	− 3.73 ± 5.09	− 5.31 ± 4.53	0.282
P*	0.013	< 0.001	
PUF Bothersome score			
1mo			
n	56	51	
Mean change from baseline	− 5.27 ± 2.61	− 4.39 ± 2.43	0.076
P*	< 0.001	< 0.001	
6mo			
n	47	39	
Mean change from baseline	− 3.60 ± 2.97	− 3.26 ± 2.90	0.595
P*	< 0.001	< 0.001	
9mo			
n	33	41	
Mean change from baseline	− 3.79 ± 2.93	− 3.66 ± 2.62	0.842
P*	< 0.001	< 0.001	
12mo			
n	38	37	
Mean change from baseline	− 3.18 ± 2.55	− 2.89 ± 3.03	0.652
P*	< 0.001	< 0.001	
18mo			
n	11	35	
Mean change from baseline	− 3.18 ± 2.68	− 3.49 ± 2.57	.736
P*	0.003	< 0.001	
24mo			
n	15	36	
Mean change from baseline	− 1.67 ± 3.35	− 3.25 ± 3.17	0.097
P*	0.135	< 0.001	
Pain VAS			
1mo			
n	56	51	
Mean change from baseline	− 5.55 ± 2.32	− 6.22 ± 2.07	0.124
P*	< 0.001	< 0.001	
6mo			
n	47	39	
Mean change from baseline	− 4.36 ± 2.82	− 5.21 ± 2.81	0.170
P*	< 0.001	< 0.001	
9mo			
n	33	41	
Mean change from baseline	− 4.58 ± 2.91	− 5.44 ± 2.61	0.183
P*	< 0.001	< 0.001	
12mo			
n	38	37	
Mean change from baseline	− 4.39 ± 2.73	− 4.76 ± 2.52	0.553
P*	< 0.001	< 0.001	
18mo			
n	9	35	
Mean change from baseline	− 3.56 ± 1.94	− 5.23 ± 2.77	0.096
P*	< 0.001	< 0.001	
24mo			
n	14	36	
Mean change from baseline	− 2.50 ± 3.03	− 4.97 ± 3.34	0.020
P*	0.009	< 0.001	

*HACS* hyaluronic acid and chondroitin sulfate; *ICSI* O’Leary–Sant Interstitial Cystitis questionnaire symptom index; *ICPI* O’Leary-Sant Interstitial Cystitis questionnaire problem index; *PUF* Pelvic pain and urgency/frequency; *VAS* visual analog scale.

*Compared to baseline, ^†^ between the two groups.

### Voiding diary parameters

Both groups showed the lowest daytime frequency, nocturia, and urgency episodes 1 month after surgery. The differences in voiding diary parameters between the control and HACS groups were not significant at any time. However, from 12 to 24 months, the degree of improvement in the daytime frequency, number of nocturia episodes, and urgency episodes in the HACS group were greater than those in the control group, and more patients showed a tendency to maintain well (Table 5).

**Table 5:** Changes in voiding diary parameters after treatment between the groups at each study visit (Per-Protocol Set).

	Control group	HACS group	*P*^†^
Daytime frequency			
1mo			
n	54	45	
Mean change from baseline	− 5.08 ± 6.85	− 5.44 ± 5.75	0.757
P*	< 0.001	< 0.001	
6mo			
n	45	34	
Mean change from baseline	− 3.07 ± 4.37	− 4.39 ± 7.60	0.751
P*	< 0.001	< 0.001	
9mo			
n	32	32	
Mean change from baseline	− 3.22 ± 3.62	− 6.19 ± 6.53	0.029
P*	< 0.001	< 0.001	
12mo			
n	32	31	
Mean change from baseline	− 2.22 ± 4.18	− 4.0 ± 5.64	0.159
P*	0.005	< 0.001	
18mo			
n	6	30	
Mean change from baseline	− 3.64 ± 3.62	− 4.57 ± 5.58	0.700
P*	0.057	< 0.001	
24mo			
n	3	23	
Mean change from baseline	− 0.55 ± 4.74	− 5.2 ± 6.28	NA
P*	NA	< 0.001	
Nocturia			
1mo			
n	54	45	
Mean change from baseline	− 1.68 ± 2.39	− 1.17 ± 1.88	0.419
P*	< 0.001	< 0.001	
6mo			
n	45	34	
Mean change from baseline	− 0.76 ± 1.63	− 0.75 ± 1.92	0.905
P*	< 0.001	0.030	
9mo			
n	32	32	
Mean change from baseline	− 0.89 ± 2.00	− 0.95 ± 2.01	0.768
P*	0.009	0.012	
12mo			
n	32	31	
Mean change from baseline	− 0.85 ± 1.81	− 1.23 ± 1.97	0.398
P*	0.012	0.002	
18mo			
n	6	30	
Mean change from baseline	− 1.53 ± 1.41	− 1.00 ± 2.44	0.615
P*	0.050	0.033	
24mo			
n	3	22	
Mean change from baseline	0 ± 2.52	− 0.98 ± 2.18	NA
P*	NA	0.047	
Urgency episode			
1mo			
n	54	40	
Mean change from baseline	− 8.55 ± 11.35	− 6.91 ± 8.93	0.652
P*	< 0.001	< 0.001	
6mo			
n	45	29	
Mean change from baseline	− 6.98 ± 9.49	− 4.6 ± 11.11	0.327
P*	< 0.001	0.034	
9mo			
n	32	27	
Mean change from baseline	− 7.28 ± 8.17	− 8.26 ± 9.76	0.675
P*	< 0.001	< 0.001	
12mo			
n	32	26	
Mean change from baseline	− 5.59 ± 8.00	− 6.59 ± 9.16	0.328
P*	< 0.001	0.001	
18mo			
n	6	25	
Mean change from baseline	− 5.91 ± 9.28	− 6.69 ± 10.45	869
P*	0.179	0.004	
24mo			
n	3	18	
Mean change from baseline	− 5.11 ± 11.08	− 6.83 ± 8.44	NA
P*	NA	0.003	

*HACS* hyaluronic acid and chondroitin sulfate; *NA* not applicable (analysis was not performed because the number of control groups was less than five).

*Compared to baseline, ^†^ between the two groups.

## Discussion

In this prospective study involving patients with Hunner type IC, the recurrence rate was significantly lower among patients who received intravesical HACS instillation treatment after transurethral ablation of the Hunner lesion than among those who underwent ablation alone. The patients' subjective symptoms showed significant improvement only with surgical treatment; however, the maintenance period of the improved condition tended to increase in the HACS group.

Although the exact etiology of IC remains unclear, Hunner lesions are characterized by severe inflammation and urothelial denudation. Recently, accumulating evidence has shown that IC/BPS without Hunner lesions and Hunner type IC are distinct pathological entities. Patients with Hunner type IC responded remarkably well to targeted endoscopic ablation of Hunner lesions. The improvement in symptoms was dramatic and persisted for approximately 12 months until the recurrence of the Hunner lesions. Interestingly, the bladder mucosa, which appears normal without Hunner lesions in patients with Hunner type IC, shows severe histological inflammation. Notably, these histological changes can be observed in the entire bladder and are not confined to the Hunner lesions^7^. Even when the recurrence pattern was prospectively observed after endoscopic ablation, most recurrences of Hunner lesions occurred in the vicinity of the ablation site, but the rate of recurrence at new sites reaches about 50%^17^. For severe pain caused by Hunner lesions, symptom relief may be maintained for a while by ablation of the Hunner lesions. However, it is still not possible to prevent the recurrence of the Hunner lesions and the progression of inflammation. Prevention of newly developed Hunner lesions will ultimately be the best way to treat IC/BPS as a severe inflammatory disease.

In this respect, GAG replacement is certainly a good treatment option for the entire bladder. IC/BPS may be related to a primary defect in the GAG layer of the urothelium and reduced expression of tight junctions^18–20^. In addition, a “cascade” of inflammatory events, which fail to be restored, may lead to chronic extracellular matrix degradation and neuroinflammation. In this regard, the restoration of the urothelium with exogenous GAG installation can help reinstate epithelial integrity in patients with IC/BPS^21–23^. However, it may not be very effective in cases of IC that already have Hunner lesions. In this study, we believe that GAG replacement, such as HACS, after surgical removal of Hunner lesions is very helpful in suppressing the progression of the Hunner lesions as well as regeneration of healthy urothelium after ablation.

Commercially available GAG-replenishing substances include heparin, HA, CS and pentosan polysulfate. CS is a glycoprotein and a component of the GAG layer of bladder mucosa. CS has been reported to play a key role in inflammation and might stimulate proteoglycan synthesis, thus reconstituting the urothelium^24^. HA is the only non-sulfated GAG that directly interacts with the cell surface^25^. This interaction may reduce urothelium permeability and stimulate sulfated GAG synthesis^26^. These agents have been used in patients who respond poorly to conventional therapies. GAG replacement has been used for a long time to treat patients with IC/BPS, but the level of evidence is low.

HA is used at a concentration of 0.8% for intravesical instillation treatment for IC/BPS. In a study in which intravesical HA was administered for 4 weeks and monthly instillation for 6 months in patients with refractory IC, the response rate at 12 weeks was 71% and was maintained well until 20 weeks^27^. In 2010 and 2012, Nickel et al.^28,29^ conducted two consecutive studies on 98 women who were administered 2% CS for 8 weeks and found no significant difference in pain and voiding symptoms at 4 weeks compared to the placebo group. Cervigni et al.^29^ randomized 110 women to receive either HACS or DMSO and evaluated the VAS for pain after 6 months. HACS group was as effective as DMSO and showed a more favorable outcome in terms of safety. Porru et al.^30^ fount that an improvement rate of 54% at 6 months after HACS instillation in 20 patients. In patients with refractory IC/BPS patients, the HACS maintained improvement in symptoms for up to 3 years, so the HACS might be more effective than the monotherapy^31^. Installing HA and CS together may offer more effective and long-lasting therapeutic advantages than either one alone.

Most studies related to HA and CS for patients with IC/BPS were conducted on women, and the studies were conducted regardless of the presence of Hunner lesions; therefore, it was not possible to distinguish between Hunner type IC or IC/BPS without Hunner lesions. In addition, the number of patients was relatively small, and the follow-up period was very short. The primary outcome of previous studies was mostly the subjective symptoms of the patient, such as pain and urinary symptoms, and there were no studies confirming the recurrence of Hunner lesions after intravesical instillation treatment. It is significant to note that we prospectively observed 78 patients with Hunner type IC in men as well as women who complained of chronic bladder pain for 2 years. In addition, periodic cystoscopy was performed to confirm the recurrence of the Hunner lesion, and subjective symptom worsening was specifically evaluated through disease-specific questionnaires.

However, this study had several limitations. First, since the incidence of Hunner type IC patients was not high, it was difficult to conduct a randomized controlled trial. To compensate for this limitation, propensity score matching was performed between the two groups. Despite these findings, caution is required when interpreting the results. Further randomized clinical trials are needed to validate these findings. Second, we defined only those patients who received 10 HACS instillations as the analysis set. This study aimed to confirm the effect of instillation treatment by maintaining the same instillation time. Some patients refused instillation treatment and were not included in the analysis set because they were not able to receive 10 intravesical instillations. One patient failed to receive the full number of HACS instillations due to recurrence, and this patient was included in the analysis. Third, if intravesical treatment, such as HACS is effective for Hunner type IC, a clear protocol on how long it should be maintained and whether it is better to use it continuously needs to be clarified in further studies.

In conclusion, among the treatment methods known to date, intravesical HACS instillation after transurethral ablation of the Hunner lesions is an effective treatment for Hunner type IC through structural and functional regeneration of the bladder as well as improvement of subjective symptoms.

## Methods

### Study design and patients

This was a single-center, prospective, single-arm study in which the treatment group was compared with a historical control group. The eligible participants were patients aged > 20 years with IC/BPS who had bladder pain with a pain for VAS of 4 or higher lasting for more than 6 months and Hunner lesions confirmed through cystoscopy, who were scheduled to undergo transurethral ablation. One month after surgery, the patients underwent intravesical HACS instillation and were followed up for 2 years (HACS group). Informed consent was obtained from all patients enrolled in this study. This clinical trial was conducted in accordance with the principles of the Declaration of Helsinki and in good clinical practice. The study was approved by the Institutional Review Board of Samsung Medical Center (approval number: 2017-08-106) and registered in ClinicalTrials.gov (NCT03463499, registration date: 13/03/2018).

The historical control group consisted of patients who were registered in our institute’s IC/BPS registry and were followed up for one year after undergoing transurethral ablation of the Hunner lesions in the previous study^15^. Since this historical control group has been further followed up, we considered the extended historical control group by applying the same inclusion and exclusion criteria and assessments as used for the HACS group (see “Supplementary” for details). Patients with a history of HACS instillation were excluded from the extended historical control group.

### Intravesical HACS (Ialuril®)

**Figure 3:**
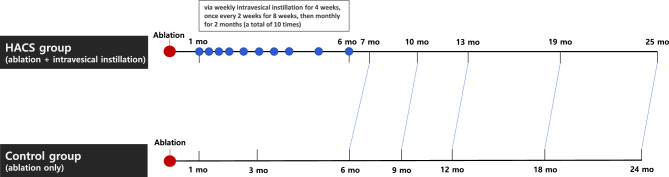
Study flow.

One month after transurethral ablation, the patient was subsequently treated with intravesical instillation therapy. Patients in the HACS group received a sterile solution containing 800 mg/50 mL sodium hyaluronic acid (HA) (1.6%) and 1 g/50 mL sodium chondroitin sulfate (CS) (2%) (HACS; iAluiRil® Prefill; IBSA, Lodi, Italy) via weekly intravesical instillation for 4 weeks, once every 2 weeks for 8 weeks, and monthly for 2 months (a total of 10 times) (Fig. 3). Patients were instructed to refrain from urinating for at least 2 h after intravesical instillation.

### Assessments

A 3-day voiding diary and self-reported questionnaires, including ICSI and ICPI, PUF symptom scale, and VAS for pain were used to evaluate the severity of symptoms at baseline and 1 month after surgery and 7, 10, 13, 19 and 25 months after surgery (i.e., 1, 4, 7, 13 and 19 months respectively, after intravesical instillation treatment). To assess recurrence, all patients underwent cystoscopy 3 months after surgery and 1 and 19 months after intravesical instillation treatment (7 and 25 months after surgery). If the patient’s symptoms aggravated, an additional cystoscopic examination was performed to confirm recurrence. All patients who visited the hospital at least once after the surgery were included in the analysis. Descriptive safety data were collected at every visit.

### End-points

The primary outcome was the recurrence of which the 2-year rate was compared between the HACS group and the historical control group. Recurrence was defined as a case in which pain returned to the baseline level and the Hunner lesion was identified on cystoscopy. Recurrence included recurrence at a previous ablation site and new lesions elsewhere in the bladder. The secondary outcomes included time-to-recurrence, changes in voiding symptoms from baseline using the 3-day voiding diary parameters, and changes in quality of life and symptom severity from baseline assessed using ICSI, ICPI, PUF, and VAS for pain. They were analyzed in the HACS and the extended historical control group.

### Statistical analyses

#### *Comparison of* the 2-year recurrence rate* between the HACS group versus the historical control group*

After transurethral ablation, the recurrence rate for 2 years was 75% according to the previous study^15^. The primary hypothesis for efficacy evaluation was that the 2-year recurrence rate after intravesical instillation of HACS would be less than that of the historical control group (i.e., 75%). The 2-year recurrence rate for the HACS group was estimated with 95% confidence interval (CI) using the Clopper–Pearson exact method and compared with the historical control group by a one-sample one-sided binomial test. For sample size calculation, we assumed that a clinically important reduction in the recurrence rate for HACS group would be at least 15%. Then, 62 participants in the HACS group were required to prove the primary hypothesis with a significance level of 5% and an expected power of 80%. Without considering the dropout rate, we enrolled patients who had received all scheduled HACS treatments until the number of participants reached 62.

#### Comparison of the recurrent-free survival between the HACS versus the extended historical control group groups

For the additional efficacy evaluation, the recurrent-free survival (RFS) was compared between the HACS and the extended historical control groups. Primary analysis was performed on the per-protocol analysis (PPA) set, along with a supplementary analysis based on the full analysis (FA) set. Definitions of the PPA and FA sets are provided in the “Supplementary Information”. Safety was evaluated in the FA set of the HACS group. Baseline characteristics were summarized by mean with standard deviation and frequency with percentage for continuous and categorical variables, respectively. Group comparisons were conducted using t-test or Wilcoxon rank sum test for continuous variables; and using chi-square test or Fisher's exact test for categorical variables, according to the satisfaction of the normality assumption.

RFS curves for the two groups were estimated using the Kaplan–Meier method and compared using the log-rank test. Multivariable Cox proportional hazards (PH) regression model was used for comparison after adjusting for potential confounders that showed statistical significance with *p*-value < 0.2 in the descriptive analysis or clinical significance among baseline characteristics. Proportional hazard assumption was checked using Schoenfeld residuals. If the assumption was violated, we used the time-dependent Cox PH regression model. The median follow-up time was estimated by the reverse Kaplan–Meier method.

All analyses were performed using the SAS software (version 9.4; SAS Institute Inc.). Statistical significance was declared with *p*-value < 0.05 unless specified.

### Supplementary Information

Supplementary Information.

## Data Availability

The datasets used and/or analysed during the current study available from the corresponding author on reasonable request.
