# Supplementary material for: Recurrence after postoperative intravesical instillation therapy in Hunner type interstitial cystitis
Source: Sci Rep. 2023 Oct 25;13:18256. doi: 10.1038/s41598-023-44894-x (PMC10600162; doi:10.1038/s41598-023-44894-x)
Supplement: Supplementary file 1 — Supplementary Information. [file 41598_2023_44894_MOESM1_ESM.docx]

**Appendix. Study design**

1. Study design

Single center, prospective single-arm study with extended historical control

2. treatment method

1. Transurethral ablation of interstitial cystitis
   1. Specific Hunner lesions in the bladder in interstitial cystitis/Chronic pelvic pain syndrome patients are resected or coagulated
2. Intravesical HACS (Ialuril^®^) instillation
   1. Patients receive intravesical injection of 1 vial (50mL) of Ialuril^®^ (1.6% Sodium hyaluronate (800mg) & 2% Chondroitin sulfate (1,000mg)) via 10F nelaton catheter, one month after receiving transurethral ablation. Patients are then instructed to refrain from voiding for two hours after receiving intravesical instillation. Patients receive intravesical instillation for a total of ten times. The instillation cycle is as follows: one instillation per week for four weeks (four times in four weeks), then one instillation per two weeks for eight weeks (four times in eight weeks), then one instillation per four weeks for eight weeks (two instillations in eight weeks).

3. Selection of study population

1) Treatment group (HACS instillation group)

- Patients receive intravesical HACS (Ialuril^®^) instillation one month after transurethral ablation of Hunner lesion, for a total of 10 instillation over five months. The patients are then followed-up for two years.

2) Extended historical control group (transurethral ablation only group)

- This group of patients consists of those who have been registered in our institute’s prospective registry, who were followed up for a total of two years after receiving transurethral ablation of HL.

This group of patients have shown about 75% recurrence rate after one year of follow-up period, which is used as the reference of recurrence rate. After excluding one patient who have opted out of the study, the total group includes 71 patients.

4. Study period

1) Treatment group (HACS instillation group)

- Screening period, followed by transurethral ablation of Hunner lesion, then were followed-up at 1 month (HACS instillation start) and at 7 months (one month after last HACS instillation), 10, 13, 19 and 25 months after surgery (can be considered to be 1, 4, 7, 13 and 19 months after intravesical instillation treatment).

2) Extended historical control group (transurethral ablation only group)

- Screening period, followed by transurethral ablation of Hunnner lesion, then were followed-up at 1, 3, 6, 9, and 12 months to determine efficacy and were then followed up at 18 and 24 months.
